# Caring for racially minoritized patients in oral medicine education: An interactive e‐resource

**DOI:** 10.1002/jdd.13487

**Published:** 2024-02-13

**Authors:** Patricia Neville, Chrysanthi Tseloudi, Konrad Spiteri Staines

**Affiliations:** ^1^ Bristol Dental School University of Bristol Bristol UK; ^2^ Digital Education Office University of Bristol Bristol UK; ^3^ Department of Oral Medicine, Bristol Dental School University of Bristol Bristol UK

## 1 PROBLEM

Since the ‘Rhodes must Fall’ student movement in South Africa in 2015 and the resultant decolonizing of the curriculum movement, higher education and academic disciplines have been grappling with the question of what constitutes knowledge. The global movement to decolonize the curriculum has positioned dental schools to critically self‐reflect on the role that power, privilege, and coloniality play in the production and reproduction of dental knowledge and the dental workforce.^1^ Dental science and curricula are now recognized as being epistemologically incomplete, with representations of racially minoritized groups and their health concerns and needs, missing, low on research agendas or misrepresented by stereotyping or over‐generalization.^2^ In the UK, dental programs are constituted by a high level of ethnic diversity, with 59% of dental students coming from ethnic minority backgrounds.^3^ This is a higher level of ethnic diversity compared to the UK.^4^ Our students routinely commented that the erasure of minoritized bodies from dental science and the over‐representation of white bodies and mouths in curricular materials are problematic. This knowledge gap is recognized as contributing to a systemic racial bias underpinning clinical teaching and reasoning, priming students and practitioners to only recognize how disease presents in white populations, contributing to cognitive bias in clinical practice.^5^ This bias can increase the risk of making diagnostic errors in diverse patient populations.^3^ Despite this, most dental teaching materials include ethnically diverse patients in a tokenistic manner, as clinical sidenotes with few clinical images.^6^

## 2 SOLUTION

Over a 6‐month period, two dental educators (K.S. [oral medicine], P.N. [sociologist]) and a learning technologist (C.T.) created an educational e‐resource for advanced dental undergraduates (i.e., year 4 of a 5‐year undergraduate dental surgery degree) to re‐center ethnically diverse patients in oral medicine instruction. Its aim was to contribute to student knowledge of, and increase their diagnostic confidence regarding the clinical presentation of oral diseases in ethnically diverse patient populations. K.S. sourced clinical images of racially minoritized patients with dark(er) skins from his and his oral medicine team's records and wrote a series of case notes to accompany these images. These clinical images were sourced and shared in compliance with hospital policy on clinical images. The Analysis, Design, Development, Implementation, and Evaluation (ADDIE) learner‐centered instructional design framework^7^ was followed. This involves identifying a problem (i.e., the underrepresentation of racially minoritized patients in oral medicine resources), taking case notes and designing 13 clinical cases (completed with a set of learning objectives, learning model adopted, and evaluation methods), developing the instructional strategies and methods and learning technologies needed to deploy the e‐resource (a case‐based learning approach, with a range of interactive activities and quizzes woven into each case study to consolidate learning and keep the learner engaged), and inviting students to use (implementation) and evaluate the e‐resource. Each case study was narrated by K.S. to improve the accessibility of the case studies. This educational e‐resource was field tested through pre‐ and post‐viewing evaluation surveys by a sample of dental students (*n* = 10) to assess its usability and effectiveness.

## 3 FINDINGS

Ninety percent of the sample “strongly agreed” that the e‐resource achieved its learning outcomes and that an interactive, case‐based teaching approach was pedagogically appropriate for this topic. This initiative inserts the oral disease experience of racially minoritized groups into the dental curricula and as such represents a diversified rather than decolonized curricular innovation. Diversifying curricula involves deliberately including the voices and experiences of non‐White populations in all curricular materials. Other diversifying strategies include expanding reading lists and citational practices used in teaching materials to include non‐Western authors.^6^ Dental schools need to adequately support and provide resources to staff designing diversified teaching resources to make sure that the creation and integration of these new materials are sustainable.

It is important to note that the educational impact of diversified teaching and curricular practices is limited because these practices alone do not make curricula inclusive. For dental education to transform its disciplinary knowledge and clinical practice and be anti‐oppressive and non‐discriminatory, more work is needed.^8^ A decolonized dental curriculum continues to critically interrogate the dental canon for biases and gaps, dismantling its power structures to create an inclusive and non‐hierarchical discipline. This can be achieved through strategic curriculum planning^9^ and the adoption of empowering pedagogies, such as active critical learning and backward teaching design.^10^ The team now plans to develop and assess the e‐resources further. Since 95% of usability problems can be identified in a sample of five to six people, a sample size of 10 was considered adequate to assess usability, but a larger sample would be needed to evaluate the e‐resource.^11^

## CONFLICT OF INTEREST STATEMENT

The authors declare they have no conflicts of interest.
